# Editorial: The Emergence and Development of Scientific Thinking During the Early Years: Basic Processes and Supportive Contexts

**DOI:** 10.3389/fpsyg.2021.629384

**Published:** 2021-02-19

**Authors:** Christopher Osterhaus, Amanda C. Brandone, Stella Vosniadou, Ageliki Nicolopoulou

**Affiliations:** ^1^Department of Psychology, Ludwig Maximillian University of Munich, Munich, Germany; ^2^Department of Psychology, Lehigh University, Bethlehem, PA, United States; ^3^College of Education, Psychology and Social Work, Flinders University, Adelaide, SA, Australia

**Keywords:** scientific reasoning, science concepts, science education, parent-child interaction, domain-general, domain-specific, inquiry-based learning, conceptual change

Scientific ways of knowing and the ability to think and act to enhance our understanding of the natural and social world are among the greatest human intellectual achievements. In modern knowledge-rich societies, scientific thinking is of crucial importance since it allows participation in increasingly complex public discourse and informed decision-making about socio-scientific issues (Sadler, 2004), such as climate change or health crises. Accordingly, many regard scientific thinking to be a critical 21st century skill which should be fostered from early in development (Trilling and Fadel, 2009).

Early developmental and educational researchers have been skeptical about young children's science competencies—including both early *science understanding* (i.e., the knowledge about scientific explanations of phenomena across various domains) and early *scientific reasoning* (i.e., the reasoning process involved in the construction of science knowledge), which together make up scientific thinking (Kuhn, 2002). In particular, research on science understanding has shown how difficult it is for children to develop scientific explanations of the world when they are in conflict with children's intuitive understandings (Vosniadou and Brewer, 1992); research on scientific reasoning has shown that young children tend to be unsystematic in the experimentation strategies they use (Tschirgi, 1980), forget to keep track of inquiry goals, experiments, and outcomes (Kuhn et al., 2008), and try to produce rather than to investigate causal effects (Lehrer and Schauble, 2007). Although difficulties persist throughout elementary school (e.g., Kuhn et al., 1995) and even among adults (Wason, 1968), recent research has shown that a broad range of scientific thinking skills are present earlier than previously expected (Sodian et al., 1991; Ruffman et al., 1993; van der Graaf et al., 2015; Köksal-Tuncer and Sodian, 2018; Koerber and Osterhaus, 2019).

The present Research Topic adds to these findings by bringing together cutting-edge research on both the basic abilities in early childhood that form the foundation of mature scientific thinking as well as the contexts, strategies, and processes that support its development. Specifically, articles in this Research Topic explore (1) the family interactions at home and in museum contexts that encourage the development of early science concepts, (2) the domain-specific and domain-general cognitive processes involved in the acquisition of scientific thinking, (3) the development and facilitation of scientific reasoning, and (4) how and under which conditions scientific thinking can promote the acquisition and revision of science knowledge.

## Family Interactions at Home and in Museum Contexts Around Science Concepts

Informal everyday interactions between parents and children are the earliest contexts that help children develop their science knowledge, taking advantage of their curiosity about the physical world, which Jirout considers critical for promoting science knowledge in young children. In recent years, there has been increased interest in these informal everyday contexts, which is also reflected in the articles in this Research Topic.

Luce and Callanan analyzed parents' everyday conversations about heat and temperature with 2–6-year-olds drawn from the CHILDES language database and from a parent-child book-reading study. This study highlights the need for detailed investigations of everyday verbal input that children receive to shed further light on how children build intuitive concepts about science phenomena.

The rest of the articles in this section focus on how to best structure parental interactions to encourage the development of science understanding, especially at informal museum science exhibits. Leech et al. provide evidence that having parents read children's books that include mechanistic explanations about science concepts to their 4- and 5-year-olds prompted these dyads to use more mechanistic language and to more successfully solve a related science problem. Similarly, Franse et al. found that when parents had pre-knowledge of a task presented in a museum exhibit by being shown the solution, they were better able to scaffold their 8–12-year-olds than parents who did not have pre-knowledge: the parents with pre-knowledge interacted longer with their children, asked more open-ended questions, led their children to inquire on their own, and were less likely to interpret the results for them than parents with no pre-knowledge. Finally, Chandler-Campbell et al. demonstrated that parents who participated in an *inquiry-based* rather than a *statement-sharing* intervention were able to better leverage children's curiosity: these parents asked more questions, including causal ones, and children provided more scientific content in response. Taken together, these studies indicate that even in informal museum contexts the best results in promoting science knowledge require parental guidance and inquiry-based scaffolding from knowledgeable parents.

## The Role of Domain-Specific and Domain-General Cognitive Processes

While in the earliest contexts, parents play an important role in promoting children's science learning, with age children become increasingly able to independently engage in scientific reasoning. Scientific reasoning is defined as intentional knowledge seeking (Kuhn, 2002). As such, developing basic scientific reasoning skills requires, first and foremost, that children recognize that knowledge is not simply a copy of the external world, but rather that knowledge claims are judgments evaluated in light of evidence (Kuhn et al., 2008). Once children attain this foundational insight, they develop a more complex understanding of how the human mind works and, in turn, are ready to acquire a more advanced understanding of the nature of science (Osterhaus et al., 2017), including the realization that scientists feel certain about their beliefs when their beliefs are supported by their interpretations of evidence.

Children's understanding of the constructive nature of knowledge has been studied extensively by researchers interested in (advanced) theory of mind and is also examined in relation to scientific reasoning in this Research Topic. In a 2-year longitudinal study involving 7–8-year-old children, Weinstock et al. found a link between children's early understanding of the interpretative nature of the mind and their epistemological understanding. That is, children who better understood that representations of the external world result from our mind's active interpretation, were more likely to apply that knowledge in a scientific context (epistemological understanding). Kyriakopoulou and Vosniadou showed that higher-order false belief reasoning (i.e., recognizing that someone may hold a false belief about a belief) in 10–12-year-olds was associated with more mature epistemological beliefs and more advanced knowledge about observational astronomy.

In addition to theory of mind, ample metacognition and better information-processing skills are also necessary to develop advanced scientific thinking. Consistent with this emphasis on domain-general cognitive processes, Betz and Coley demonstrated that conceptual flexibility (i.e., the ability to switch between different ways of organizing knowledge) increased with both age and experience, with implications for children's biological knowledge. Young and Shtulman reported that 5–12-year-olds' cognitive reflection (defined as the tendency to reflect on one's own thinking) was related to children's understanding of counterintuitive science ideas. And Fridman et al. demonstrated the impact of metacognition and self-regulation on 5–6-year-olds' scientific exploration. Taken together, these studies highlight the ways in which cognitive abilities outside the realm of science (e.g., metacognition, self-regulation, conceptual flexibility, theory of mind) are associated with children's ability to reason scientifically.

## Development and Facilitation of Diverse Forms of Scientific Reasoning

Children's exploration of science phenomena has primarily been investigated by asking how proficient they are in experimentation using the control-of-variables strategy (CVS). CVS holds that informative experiments must only vary a single variable at a time while keeping all others constant, and as such, allows for systematic exploration of cause-effect relations. A consistent finding in the literature is that CVS is difficult for elementary school children (e.g., Kuhn et al., 1995; Bullock and Ziegler, 1999; Croker and Buchanan, 2011). However, experimentation is not the only mode of scientific reasoning (Kind and Osborne, 2017), and studies in this Research Topic highlight several other important forms of scientific reasoning, including some that develop much earlier than the ability to control variables.

Klemm et al. found that 4–6-year-olds have competencies in observation that go beyond merely making observations and include diverse epistemic activities, such as asking questions about, testing, or making sense of observations. Weisberg et al. demonstrated that 4–10-year-olds are capable of diagnostic reasoning (i.e., the ability to infer causes from systematic observations of patterns of data about cause-effect relations) across multiple contexts. Datsogianni et al. reported abilities in conditional reasoning (i.e., reasoning about if-then statements) in both familiar and mathematical contexts in children aged 7–12 years. And Peteranderl and Edelsbrunner demonstrated important precursors to the development of CVS in 9–11-year-olds, including an understanding of indeterminacy (whether available evidence is sufficient to warrant a conclusion) and confounding (whether confounding variables are appropriately controlled). In sum, although observation, data interpretation, conditional or diagnostic reasoning, and experimentation may vary in complexity and age of acquisition, the studies in this section highlight how each of these processes can be considered genuine forms of scientific reasoning.

Importantly, Schlatter et al. showed that these abilities are also responsive to intervention. In particular, a 5-week training program resulted in significant competence gains across diverse scientific reasoning abilities (i.e., hypothesis formation, experimentation, and data interpretation). Whether or not these effects are long-lasting, and whether individual differences in children's performance on different scientific reasoning measures are stable over time, remain open questions.

## Scientific Thinking in the Service of Promoting Changes in Science Knowledge

The scientific reasoning processes described above can be applied in the service of the acquisition of science knowledge. A large body of research has shown that children have intuitive knowledge in various science domains, including physics, biology, and astronomy (Shtulman and Walker, 2020), and that they bring this intuitive knowledge to the task of science learning (Vosniadou, 2019). Thus, the development of accurate science knowledge often involves a gradual process of assimilating new knowledge and revising or replacing prior scientifically-incorrect beliefs (Vosniadou, 2002). Several papers in this Research Topic examine these processes, shedding light on the conditions under which the revision of science knowledge takes place and how it can be promoted and assessed.

Van Schaik et al. found that providing systematic evidence highlighting key variables (vs. non-systematic evidence) promoted 4–9-year-olds' predictions and explanations about buoyancy events. Hardy et al. showed that conditions designed to facilitate comparison also enhanced 4–7-year-olds' predictions about buoyancy. Larsen et al. and Weber et al. explored how the nature of the evidence children receive impacts revisions to their science knowledge. Larsen et al. found that 5-year-olds' learning about balance was facilitated following anomalous evidence experienced directly (through a hand-on task) and indirectly (through illustrations in a picture book) compared to a control condition. Moreover, Weber et al. found that children who received a combination of both verbal and material forms of scaffolding in a play-based intervention were most likely to adjust their theories about balance in the face of counterevidence. Van der Graaf further demonstrated how an inquiry-based lesson involving generating hypotheses and gathering and evaluating evidence promoted conceptual change as revealed by changes in 8–13-year-olds' strategy use on a balance beam task. Finally, Gaudreau et al. found that the gestures third-graders use when describing the day/night cycle can provide insight into their developing science knowledge by reflecting children's current understandings and potentially foreshadowing future conceptual change.

Findings from across these studies reveal advances and revisions in children's science knowledge that are driven by salient and often hands-on learning experiences. Children can engage in belief revision when they are confronted with systematic evidence (including evidence that conflicts with their prior beliefs) and when they are encouraged to reflect on or explain that evidence. Consistent with the above findings about parent-child interactions and the broader literature on inquiry-based learning (e.g., Lazonder and Harmsen, 2016), the studies in this section also suggest that learning and belief revision are more successful when the task includes greater structure and specific guidance. Finally, these studies highlight the various ways in which children's initial knowledge can facilitate or constrain their ability to learn from interventions.

## Concluding Remarks And Future Directions

The wide range of studies in this Research Topic from both developmental psychology and education suggest that young children show more advanced scientific thinking than previously thought; however, further development is needed to give rise to mature science knowledge and scientific reasoning. This complex process of development involves interactions between domain-general and domain-specific cognitive processes and abilities (such as epistemological understandings, theory of mind, metacognition, self-regulation) and environmental inputs (such as informal interactions at home and museums, and formal education and instruction), which are just beginning to be understood. The articles included in this Research Topic provide a sample of the range of current research that investigates these complex interactions and provide a road map to the development of scientific thinking that is helpful and illuminating for both theory and practice.

An important task for future research will be to determine how the basic abilities discussed in the articles included in this Research Topic come together with domain-general and domain-specific cognitive processes to influence mature scientific thinking. Although there is evidence to suggest a certain degree of stability in scientific reasoning from middle childhood to adulthood (Bullock et al., 2009), more work is needed to connect mature scientific thinking with the basic science abilities present in younger children. Studies of this sort will be challenging given their longitudinal nature and the need for designing more appropriate measures of broad scientific reasoning abilities [see Koerber and Osterhaus (2019), van de Sande et al. (2019), and Osterhaus et al. (2020); for recent examples]. However, this work is important both from a theoretical perspective and in order to speak to the long-term value of efforts to foster scientific thinking in early education (Klahr et al., 2011).

In addition to documenting that young children can, in principle, explore science concepts systematically, the current Research Topic also speaks to various ways in which children's scientific thinking can be scaffolded. In particular, several studies show that young children need guidance in their systematic exploration of evidence, which is true across diverse contexts ranging from the classroom, to the science museum, to children's homes. Accordingly, designers of learning environments for young children need to consider both the type and amount of support that is needed by children of different ages and cognitive abilities. Offering too little support may result in children's failure to learn; in contrast, offering inappropriate or too much structure may diminish children's curiosity. Moving forward, it will be important to bring together researchers in developmental psychology and education–as is done in this Research Topic–to create effective opportunities to foster the critical 21st century skill of scientific thinking in the youngest members of society.

## Author Contributions

All editors contributed in substantial ways to the conception of the topic as well as in overseeing the reviewing process and to a genuinely collaborative writing of the editorial. CO and AB played an especially key role in the process. AN played a key role in bringing this team of editors together and overseeing the process. All authors contributed to the article and approved the submitted version.

## Conflict of Interest

The authors declare that the research was conducted in the absence of any commercial or financial relationships that could be construed as a potential conflict of interest.
